# Tobacco use perceptions and intentions to abstain among adolescents in the English-speaking Caribbean: A cross-sectional secondary analysis

**DOI:** 10.18332/tpc/217323

**Published:** 2026-05-30

**Authors:** Rita O. Chukwu, Maryam Hadi, Ava G. Reynolds, Qingchun Jin, Shandey D. Malcolm

**Affiliations:** 1 Department of Public Health, Purdue University, West Lafayette, United States

**Keywords:** Caribbean, adolescents, Health Belief Model, tobacco use perceptions, abstinence intentions

## Abstract

**INTRODUCTION:**

Tobacco use among Caribbean adolescents surpasses global averages. Given the adverse effects, regional efforts have prioritized reduction and eventual elimination and have adopted the World Health Organization's Framework Convention on Tobacco Control (WHO-FCTC). Guided by the Health Belief Model, we examine changes in tobacco abstinence and abstinence-related perceptions before and after the WHO-FCTC.

**METHODS:**

We conducted a cross-sectional secondary analysis of Global Youth Tobacco Survey data. We specifically examined adolescents aged 13–15 years from three Caribbean countries, including participants surveyed in 2000 (n=3431) and 2017–2018 (n=3767). We first conducted chi-squared tests and t-tests to examine changes in tobacco abstinence and abstinence-related perceptions across survey years. We then used the latest survey data (2017–2018) and logistic regression to assess associations between perceptions and intentions to abstain in the next year, among adolescents who did not report tobacco use (n=3050). All analyses incorporated survey weights and clusters.

**RESULTS:**

Compared with 2000, adolescents surveyed in 2017–2018 had improved 30-day tobacco abstinence, including cigarette abstinence (89.67% to 95.39%, p<0.01). However, intentions to abstain in the next year declined over the study years (93.65% to 90.53%, p<0.01). Key tobacco abstinence-related perceptions, including perceived severity (88.51% to 83.87%, p<0.01) and refusal self-efficacy (91.08% to 88.67%, p<0.01), also declined. In regression analysis, perceived benefits of anti-tobacco campaigns (AOR=2.25; 95% CI: 1.40–3.60) and refusal self-efficacy (AOR=11.35; 95% CI: 7.55–17.04) were associated with higher intentions to abstain. Exposure to anti-tobacco messaging (AOR=0.52; 95% CI: 0.30–0.90) was negatively associated with abstinence intentions.

**CONCLUSIONS:**

Given that abstinence-related perceptions declined across survey years and were associated with tobacco use intentions, these findings underscore the importance of continued attention to WHO-FCTC anti-tobacco initiatives that address tobacco-related perceptions. Incorporating culturally sensitive messaging may enhance the relevance and effectiveness of such efforts.

## INTRODUCTION

In the Caribbean, the prevalence of current 30-day tobacco use among adolescents (13.5%) is higher than the global average of 10.7%^1^. In recent years, use has declined, with ever smoking dropping from 33.3% to 29.0% between 2000 and 2008, and current smoking decreasing from 12.1% to 11.7% over the same period^2^. Unfortunately, the progress toward reduction has been slow and has fallen short of the goal of ‘A 100% Smoke-Free Caribbean’ by 2022^3^. The higher-than-average tobacco use among Caribbean adolescents, coupled with limited progress towards achieving reduction targets, highlights a continuing public health need in this population, especially given the associated health outcomes. Globally, tobacco use accounts for nearly seven million preventable deaths annually and is the number one actual cause of death worldwide^4^.

The adverse effects of tobacco use are even greater when initiation occurs in adolescence. Early initiation increases the odds of mental health problems, such as depression and anxiety^5^. Early initiation of tobacco use also significantly increases the risk of developing nicotine addiction, the likelihood of continued use in adulthood, and a range of adverse health outcomes that manifest later in life, including respiratory diseases, cardiovascular conditions, and metabolic disorders^6^. Moreover, adolescent tobacco consumption can interfere with vital brain areas that play crucial roles in decision-making and memory retention. This interference can result in cognitive impairments that negatively affect assimilation of educational materials among adolescents, memory function, and overall academic performance^7^. Given the numerous adverse health outcomes, several researchers and institutions have called for a focus on promoting abstinence in adolescence^8^.

In 2003, the World Health Organization (WHO) introduced the Framework Convention on Tobacco Control (WHO-FCTC). This evidence-based health promotion treaty aims to control the global tobacco crisis through a series of policies and measures^4^. The five primary demand-reduction measures include monitoring tobacco use, implementing smoke-free policies, offering cessation support, mandating health warnings, banning tobacco advertising, promotion, and sponsorship (TAPS), and increasing tobacco taxes^9,10^. Since 2005, all member states of the Caribbean Community (CARICOM), except for Haiti, have been parties to the WHO-FCTC. Countries that have fully implemented the WHO-FCTC have seen significant reductions in smoking prevalence^9^. Positive impacts have also been demonstrated among Caribbean adolescents 1–5 years after implementation, although there has been limited subsequent attention to changes in smoking prevalence^2^. Moreover, studies examining the effects of the WHO-FCTC focus solely on behavioral changes, which constitute only part of the overall picture, with less attention to key psychosocial factors which can be important to tobacco prevention^11^. Thus, beyond the limited evidence on long-term (aged >10 years) changes in abstinence, there is also scarce evidence on shifts in abstinence-related perceptions among Caribbean adolescents, leaving important gaps in understanding how tobacco-abstinence–relevant beliefs evolve over time since the WHO-FCTC.

The Health Belief Model (HBM) has been successfully used to explain positive tobacco use behaviors such as cessation and abstinence^12^. Thus, the framework may help to explore tobacco-related beliefs before and after the WHO-FCTC and may offer insights into abstinence promotion among Caribbean adolescents. According to Rosenstock et al.^13^, HBM comprises six essential elements: perceived susceptibility, perceived severity, perceived benefits, perceived barriers, cues to action, and self-efficacy. Perceived severity is an individual’s belief about the seriousness of a health condition^14^. Adolescents who perceive smoking as not harmful are more likely to use cigarettes and cigars, initiate at younger ages, and smoke more frequently^15^. Perceived barriers are an individual’s beliefs about obstacles that may hinder their ability to adopt a particular health behavior^14^. Positive outcome expectancy, that is, associating tobacco use with positive attributes such as looking ‘cool’ or ‘fitting in’, is a significant predictor of smoking initiation and intentions to smoke, and therefore can be conceived as a barrier to abstinence^16,17^.

Perceived benefits may refer to a person’s beliefs about both the positive behavioral change and various courses of action in promoting that change^14,18^. Several perceived benefits of abstinence have been identified among adolescents, including improved appearance, better hygiene, and increased social approval^19,20^. Adolescents who endorse these beliefs are more likely to abstain from smoking^19,21^. Similarly, adolescents who support regulatory efforts are less likely to use tobacco^22^. Self-efficacy is an individual’s confidence in their ability to perform, adopt, or maintain a given task or health behavior^13^. Several studies have shown that self-efficacy concerning tobacco refusal plays a key role in shaping adolescents’ intentions to avoid smoking, intention to quit smoking, success in quitting tobacco, and smoking rates^23,24^. Finally, cues to action, which are the necessary stimuli that trigger an individual to adopt a particular health behavior, are predictors of smoking avoidance^14,25^.

With a focus on prevention, our study aimed to explore tobacco abstinence and abstinence-related beliefs among Caribbean adolescents before and at least 10 years after the implementation of the WHO-FCTC, using the HBM as a framework. Changes in perception can be an early indicator of long-term behavior and, therefore, relevant to evaluating the effectiveness of the anti-tobacco campaign. To further reinforce the importance of targeting tobacco perceptions, we also sought to examine how tobacco abstinence-related beliefs influence adolescents’ intention to abstain from tobacco use in the following year. High intentions and willingness to smoke are strong predictors of smoking initiation among adolescents^26^.

## METHODS

### Study participants

We conducted a secondary analysis of publicly available Global Youth Tobacco Survey (GYTS) data from two survey waves (2000 and 2017–2018) and used cross-sectional analyses to: 1) compare tobacco-related outcomes across years, and 2) assess the association between tobacco abstinence perceptions and intentions to abstain. The GYTS is a tool developed jointly by the World Health Organization (WHO) and the Centers for Disease Control and Prevention (CDC) to monitor youth tobacco use worldwide. It utilizes a standardized methodology across countries, including a two-stage cluster sampling procedure to produce nationally representative samples of students enrolled in Grades 7 through 12. The survey was self-administered to students during a regular classroom period using a standardized questionnaire. No identifying information was collected.

**Figure 1:**
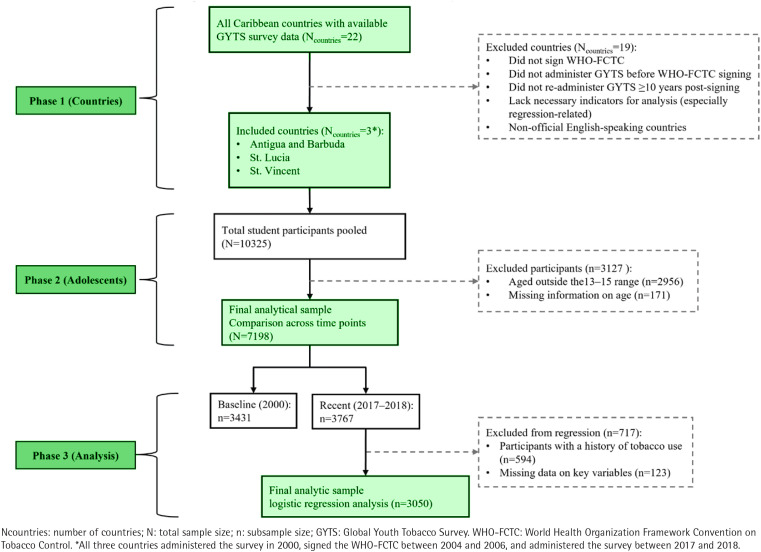
Flowchart of the selection of Caribbean countries and adolescents aged 13–15 years included in the study, a cross-sectional analysis of the Global Youth Tobacco Survey (GYTS), Caribbean region, 2000 and 2017–2018 (N=7198)

To compare tobacco-related perceptions before and after the adoption of the WHO-FCTC, we analyzed data from Antigua and Barbuda, St. Lucia, and St. Vincent. These were the three Caribbean countries that: 1) administered the survey before signing the WHO-FCTC; 2) signed the WHO-FCTC; 3) administered the GYTS again at least 10 years later; and 4) included the necessary items for the analysis, particularly for the regression analysis. We further limited the study to adolescents aged 13–15 years, the primary focus of the WHO GYTS and most regional reports. This resulted in a final analytic sample of n=3431 in 2000 and n=3767 in 2017–2018. Table 1 provides the number of respondents and participation rates for each country in both 2000 and later (2017–2018). Finally, to understand the association between tobacco-related perceptions and intentions to abstain, we utilized only the 2017–2018 data and limited the sample to adolescents who reported never using tobacco and who provided a response to all the relevant study items (n=3050). Figure 1 depicts the selection of countries and adolescents, including eligibility criteria, exclusions, and final analytic samples across survey years. Ethical approval to use the publicly available datasets was obtained from the Institutional Review Board of the senior author’s institution (IRB-2025–576).

**Table 1:** Number of respondents and participation rates from the Global Youth Tobacco Survey (GYTS) among adolescents aged 13–15 years in three English-speaking Caribbean countries, 2000 and 2017–2018

*Country*	*First Survey*			*WHO-FCTC Signature year*	*Second Survey*		
	*Year*	*N*	*Participation rate %*		*Year*	*N*	*Participation rate %*
Antigua and Barbuda	2000	1183	91.7	2004	2017	1538	87.1
St. Lucia	2000	1068	86.2	2005	2017	1234	84.3
St. Vincent	2000	1180	78.4	2004	2018	995	85.1

N: unweighted sample size. Participation rate: percentage of eligible students who completed the survey.

### Study measures

We considered two demographic characteristics: sex (male or female) and weekly spending money (recoded to indicate whether discretionary funds were available).

### Health Belief Model perceptions

We examined tobacco-related perceptions through constructs derived from the Health Belief Model (HBM). We measured perceived severity with a single item asking whether participants believed that smoking tobacco is harmful to health, with ‘Definitely yes’ and ‘Probably yes’ coded as yes (high perceived severity) and ‘Definitely no’ and ‘Probably no’ coded as no (low perceived severity). We also assessed perceived barriers with a single item: ‘Think I might enjoy smoking a cigarette’. Agreement (strongly or somewhat agree) was coded as ‘yes’, while disagreement (strongly or somewhat disagree) was coded as ‘no’, indicating the presence or absence of the cognitive barrier. We evaluated perceived benefits of tobacco abstinence using three items that asked about the influence that tobacco has on the number of friends, attractiveness, and comfort in social settings. For each item, responses indicating a negative perception of tobacco use (i.e. ‘less friends’, ‘less attractive’, ‘less comfortable’) were coded as yes, and others as no. Thus, items were coded to reflect the benefits of abstinence. A composite mean score (ranging from 0 to 1; higher values represent greater perceived benefits of abstinence) was created to represent this construct (α=0.46). Additionally, we measured the perceived benefits of tobacco control campaigns using three items assessing support for banning smoking at outdoor events, indoors, and prohibiting tobacco sales to minors. Responses of ‘Yes’ were coded as 1, and ‘No’ as 0. These were averaged to create a composite index of perceived support for anti-tobacco regulation; higher values indicated greater perceived benefits of anti-tobacco campaigns (α=0.65).

We assessed perceived self-efficacy using the item: ‘If one of your best friends offered you a tobacco product, would you use it?’ Adolescents who indicated they would ‘Definitely not’ or ‘Probably not’ accept, were considered to have high refusal self-efficacy. In contrast, those selecting ‘Definitely yes’ or ‘Probably yes’ were considered to have low refusal self-efficacy. Finally, we derived cues to action from three items related to exposure to tobacco control messaging. These included whether respondents had seen or heard anti-tobacco messages at public events, warnings on cigarette packaging, or media campaigns. Each affirmative response was coded as 1, each negative response as 0. We then created a composite mean score to quantify overall exposure, with higher values indicating greater exposure to antitobacco messages. Perceived susceptibility was excluded because there were no GYTS items that adequately captured this construct.

### Past 30-day tobacco abstinence

We utilized the standard GYTS questions on 30-day use of smoked products to measure current tobacco abstinence. Adolescents were asked whether, during the past 30 days, they had smoked any cigarettes and whether, during the past 30 days, they had used any other smoked tobacco products (e.g. cigars, pipes, or waterpipes). For each item, we coded responses indicating no past 30-day use as abstinence from that product and all other responses as use of that product.

### Next 12-month tobacco abstinence intention

Finally, we measured intention to abstain from tobacco use using a single item: ‘At any time during the next 12 months, do you think you will use any form of tobacco?’. We dichotomized responses such that ‘Definitely no’ and ‘Probably no’ were coded as intention to abstain, and ‘Probably yes’ and ‘Definitely yes’ were coded as intention to use.

### Analytic plan

We began by conducting descriptive analyses to summarize participant demographics, tobacco abstinence, and individual HBM perception items across 2000 and 2017–2018. We then conducted bivariate analyses (chi-squared tests for categorical variables and t-tests for continuous variables) to determine whether there were significant differences across the two time points. Finally, to evaluate the adjusted associations between HBM constructs and intention to abstain, we conducted a multivariable logistic regression model that included all five constructs, controlling for sex and weekly spending money. When applicable, we utilized the composite measures for HBM constructs. All analyses accounted for the GYTS complex survey design by incorporating sampling weights, clustering, and stratification using Stata version 18. Participants with missing responses on key predictors or outcome variables were excluded using complete-case analysis, consistent with standardized Stata procedures. Statistical significance was determined at p<0.05.

## RESULTS

### Participant characteristics

In 2000, 56.2% of adolescents were female, 43.8% were male, and 61.0% reported weekly spending money. Between 2016 and 2020, 49.7% of respondents were female, 50.3% male, and 88.6% reported spending money weekly. There were significant differences in sex distribution, and adolescents in the later surveys were more likely to report spending money (data not shown).

### Comparison of tobacco-related construct changes: 2000 vs 2017–2018

Table 2 compares tobacco-related perceptions, abstinence, and abstinence intentions across survey periods. Perceived severity remained high but declined significantly over time, with 88.51% in 2000 and 83.87% in 2017–2018 reporting that smoking was harmful (χ^2^=32.15, p<0.01). Perceived benefits of abstinence also decreased significantly. In 2000, 47.81% believed that smokers had fewer friends compared to 29.61% in 2017–2018 (χ^2^=173.20, p<0.01). Similarly, 83.72% viewed smokers as less attractive in 2000 compared with 63.00% in the later surveys (χ^2^=367.76, p<0.01). While there was no comparison data for 2020, only 25.6% in 2017–2018 reported that smoking made people less comfortable at social gatherings. In 2000, 74.89% supported bans on smoking in enclosed public places compared with 74.03% in 2017–2018, indicating a minor, non-significant change. Additionally, from later surveys, 76.5% supported bans on sales to minors and 66.6% endorsed bans on smoking in outdoor spaces (no comparative data are available for 2000).

**Table 2:** Tobacco-related perceptions and abstinence outcomes among adolescents aged 13–15 years in three English-speaking Caribbean countries, a cross-sectional analysis of Global Youth Tobacco Survey (GYTS) data from 2000 (N=3431) and 2017–2018 (N=3767)

*HBM Construct*	*Question*	*Response option*	*2000 %*	*2016–2020 %*	*χ² (df)*	*p*
Perceived severity	Do you think smoking tobacco is harmful to your health?	Probably/definitely yes	88.51	83.87	32.15	<0.01
Perceived benefits of abstinence	**Do you think…**					
	Young people who smoke have more or fewer friends?	Less friends	47.81	29.61	173.20	<0.01
	Smoking cigarettes or tobacco makes young people look more or less attractive?	Less attractive	83.72	63.00	367.76	<0.01
	Smoking tobacco helps people feel more comfortable or less comfortable at social gatherings?	Less comfortable	N/A	25.60	N/A	
Perceived benefits of anti-tobacco campaigns	Are you in favor of banning smoking at outdoor public places?	Yes	N/A	66.60	N/A	
	Are you in favor of banning smoking inside enclosed public places?	Yes	74.89	74.03	0.68	0.63
	Do you think the sale of tobacco products to minors should be banned?	Yes	N/A	76.50	N/A	
Perceived barriers	Do you agree or disagree with the following: I think I might enjoy smoking a cigarette	Yes	N/A	7.77	N/A	
Self-efficacy	If one of your best friends offered you a tobacco product, would you use it?	Definitely/Probably no	91.08	88.67	11.49	0.01
Cues to action	**During the past 30 days, did you…**					
	See or hear any anti-tobacco media messages on TV, radio, internet, billboards, etc.?	Yes	79.60	39.78	312	<0.01
	See or hear any anti-tobacco messages at sports, social gatherings?	Yes	70.20	31.20	375.47	<0.01
	See any health warnings on cigarette packages?	Yes	N/A	43.00	N/A	
Current tobacco abstinence	During the past 30 days, did you…					
	Smoke any cigarettes?	No	89.67	95.39	78.96	<0.001
	Use other smoked tobacco products?	No	90.32	95.48	66.99	<0.001
Tobacco abstinence intentions	At any time during the next 12 months do you think you will use any form of tobacco?	Definitely/Probably No	93.65	90.53	23.93	<0.01

Percentages are weighted estimates. χ²: design-adjusted chi-squared test. HBM: Health Belief Model. N/A: item not included in the 2000 GYTS module.

Refusal self-efficacy declined slightly but significantly, from 91.08% in 2000 to 88.67% in 2017–2018 (χ^2^=11.49, p=0.01). Cues to action also declined significantly: In 2000, 79.60% reported exposure to anti-tobacco media messages compared with 39.78% in the later surveys (χ^2^=312.00, p<0.01), and exposure to anti-tobacco messages at sports or social gatherings declined from 70.20% to 31.20% (χ^2^=375.47, p<0.01). By 2017–2018, 43.0% reported seeing health warnings on cigarette packages. Thirty-day abstinence increased significantly, while intention to abstain significantly declined. Specifically, Cigarette abstinence rose from 89.67% in 2000 to 95.39% in 2017–2018 (χ^2^=78.96, p<0.001). Abstinence from other smoked tobacco products also improved from 90.32% to 95.48% (χ^2^=66.99, p<0.001). However, intention to abstain declined from 93.65% in 2000 to 90.53% in 2017–2018 (χ^2^=23.93, p<0.01).

**Figure 2:**
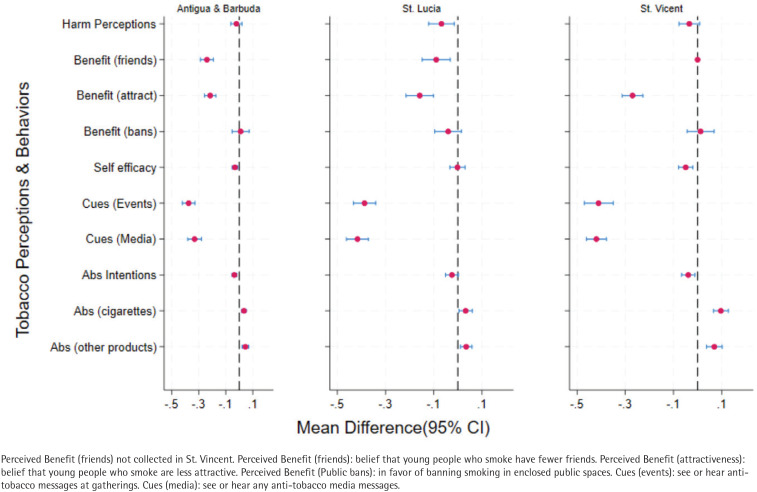
Country-level differences in tobacco-related perceptions and abstinence outcomes among adolescents aged 13–15 years in three English-speaking Caribbean countries, a cross-sectional analysis of Global Youth Tobacco Survey (GYTS) data from 2000 (N=3431) and 2017–2018 (N=3767)

Figure 2 shows changes in tobacco-related perceptions and abstinence outcomes between 2000 and 2017–2018 across the three countries. Perceived severity declined across countries, but the difference was significant only in Saint Lucia (mean difference= -0.07; 95% CI: -0.12 − -0.02). Perceived benefits of tobacco abstinence declined significantly in all countries with the largest decline related to the belief that tobacco use reduces attractiveness in Saint Vincent (mean difference= -0.27; 95% CI: -0.31 − -0.23). Refusal self-efficacy declined significantly in Antigua and Barbuda (mean difference= -0.03; 95% CI: -0.05 − -0.01) and Saint Vincent (mean difference= -0.05; 95% CI: -0.08 − -0.02), but not Saint Lucia. Reductions in cues to action were significant in all countries; the largest reduction was observed in St. Vincent (mean difference= -0.42; 95% CI: -0.46 − -0.38) in relation to seeing or hearing antitobacco messages through the media. Abstinence intentions declined in all countries, but the difference was significant only in Antigua and Barbuda (mean difference= -0.04; 95% CI: -0.05 − -0.02) and St. Vincent (mean difference= -0.04; 95% CI: -0.05 − -0.02). On the other hand, tobacco abstinence increased significantly across settings; the greatest increase was observed in relation to cigarette abstinence in St. Vincent (mean difference=0.10; 95% CI: 0.07−0.13). As noted, reductions were greatest for the cues to action and perceived benefits of abstinence items. The country-level mean differences and 95% confidence intervals are provided in Supplementary file Table 1.

### Multivariable logistic regression

Table 3 showcases how tobacco-related perceptions predict intention to abstain from tobacco among adolescents (2017–2018). Specifically, adolescents who agreed with the belief that they might enjoy smoking were less likely to intend to abstain (AOR=0.49; 95% CI: 0.32–0.74). On the other hand, higher perceived benefits of anti-tobacco campaigns (AOR=2.25; 95% CI: 1.40–3.60) were positively associated with abstinence intentions. Additionally, adolescents who reported they would refuse a tobacco offer from a friend were nearly 11 times more likely to intend to abstain (AOR=11.35; 95% CI: 7.55–17.04). Unexpectedly, cues to action (AOR=0.52; 95% CI: 0.30–0.90) were negatively associated with abstinence intentions. Specifically, those who saw anti-tobacco messages in the media or at social gatherings were less likely to intend to abstain. Perceived severity, perceived benefits of abstinence, sex, age group, and weekly spending money were not statistically significant predictors (p>0.05).

**Table 3:** Logistic regression analysis predicting intention to abstain from tobacco among adolescents aged 13–15 years in three English-speaking Caribbean countries, Global Youth Tobacco Survey (GYTS), 2017–2018 (N=3050)

*Variable*	*B*	*SE*	*AOR*	*95% CI*
Sex (Female)	-0.12	0.19	0.88	0.60–1.30
Has spending money	0.09	0.28	1.09	0.62–1.91
Perceived severity (Yes)	0.30	0.27	1.35	0.80–2.30
Perceived barrier (Agree)	-0.72	0.21	0.49**	0.32–0.74
Self-efficacy (Yes)	2.43	0.21	11.35**	7.55–17.04
Cues to action	-0.65	0.28	0.52**	0.30–0.90
Benefits of campaign	0.81	0.30	2.25**	1.40–3.60
Benefits of abstinence	-0.17	0.19	0.85	0.47–1.53

AOR: adjusted odds ratio. SE: standard error. Model adjusted for sex, age group, and weekly spending money. All analyses incorporate GYTS sampling weights, clustering, and stratification.

* p<0.05,

** p<0.01,

*** p<0.001.

## DISCUSSION

Guided by the Health Belief Model (HBM), our study explored tobacco abstinence and abstinence-related beliefs among adolescents in three Caribbean countries before and after they signed and ratified the WHO Framework on Tobacco Control (WHO-FCTC). We found an increase in abstinence beyond the 1- to 5-year post-WHO-FCTC assessment of Hambleton et al.^2^. In that study, the 30-day cigarette use among adolescents aged 13–15 years was found to be 3.4%, 12.11%, and 11.2% between 2004 and 2007 in Antigua and Barbuda, St. Lucia, and St. Vincent, respectively^2^. We found rates of 1.4%, 6.4%, and 4.3% in the same three countries during 2017–2018. However, we did not find a similar favorable change in tobacco perceptions following the WHO-FCTC, which is notable, as abstinence perceptions were associated with the intention to use tobacco the following year. Our findings provide descriptive, cross-sectional insight that may help policymakers, health education specialists, and public health practitioners contextualize WHO-FCTC anti-smoking initiatives as the region works toward a 100% smoke-free Caribbean^3^.

Adolescents were less likely to perceive tobacco as being harmful in the most recent surveys, suggesting an educational need for more contemporary adolescents. However, we also found that perceived severity was not significantly associated with tobacco abstinence intentions. These findings are consistent with the Steinberg^27^ Social Neuroscience theory, which suggests that adolescents perceive long-term risks as less relevant and often attribute a sense of invincibility to themselves. It is also consistent with another study by Panahi et al.^28^ which did not find an association between perceived severity and smoking preventive behaviors. Thus, when it comes to perceived severity, health warnings may be insufficient for adolescents. A focus on other social and contextual drivers (peer pressure, family use, desire for social status, stress coping) that can outweigh those beliefs is also needed^29,30^. We also found that adolescents were less likely to uphold positive beliefs about anti-tobacco initiatives and less likely to report tobacco refusal self-efficacy in the more recent surveys, and that these perceptions predicted intentions to abstain in the following year. Our findings suggest room for improvement in anti-tobacco initiatives, particularly in emphasizing the interpersonal and image benefits of remaining smoke-free. It also indicates the need to cultivate confidence and refusal skills in adolescents to better equip them to believe they can resist peer pressure. Several approaches have proven effective, including role-playing to practice peer modeling and positive reinforcement^31^.

An improvement in tobacco abstinence without a concurrent rise in positive abstinence-related perceptions may be related to policies that successfully limit access to tobacco products among adolescents but fail to maintain negative attitudes toward tobacco in the long-term. Other reasons for the lapse may relate to the recent rise of different forms of nicotine dispensing products such as e-cigarettes and hookah, which adolescents perceive to be less harmful, less unattractive, and more socially acceptable highlighting the need for new messaging about the risk of these products, particularly among adolescents^32^. Unfortunately, adolescents in the most recent surveys were less likely to see or hear anti-tobacco messages compared to in 2000. This trend is consistent with global developments; anti-tobacco messaging was particularly strong in the late 1990s and 2000s but has declined in recent years^33^. We paradoxically found that cues to action were associated with lower intentions to abstain from tobacco use. This may mean the current approach is eliciting a response contrary to its purpose, and may need to be reworked. While most studies show a positive influence of anti-tobacco campaigns, one in the US related to the ‘Think. Don’t Smoke’ campaign has also found antithetical effects^34^.

### Strengths and limitations

Our study findings should be considered in light of limitations. We used secondary data, which limits our ability to measure each HBM construct ideally. For instance, single-item measures may not fully capture the depth of perceived severity and perceived barriers, and were not assessed for internal consistency. We also acknowledge that the internal consistency estimates for the composite scales (α=0.46 and 0.65) are modest. However, both scales are composed of only three items, and Cronbach’s alpha is known to be sensitive to scale length, often underestimating reliability for very short measures. Additionally, this study provides no indication of the extent of WHO-FCTC implementation in each country, as documented in WHO monitoring reports. The use of a cross-sectional dataset limits the ability to draw causal or temporal conclusions about the relationship between health beliefs and intentions to abstain from tobacco use. Additionally, findings may also have limited generalizability, as the sample was restricted to adolescents aged 13–15 years from three English-speaking Caribbean countries. Finally, residual confounding, including unmeasured exposure to e-cigarettes or other nicotine products, as well as potential recall and social desirability bias inherent in self-reported survey data, should be considered when interpreting the results.

Despite this, our study has several strengths; the use of the Health Belief Model (HBM) provides a robust framework for examining the psychological factors that influence the intention to abstain from tobacco, thereby strengthening the conceptual validity and interpretability of the findings. Additionally, this study uses extensive, multi-country data from three Caribbean countries, thereby enhancing the generalizability of its findings within the region. Caribbean adolescents are disproportionately affected by tobacco-related risk factors; hence, this study addresses a critical gap in global public health research by focusing on a high-risk and understudied population.

### Implications

This study suggests a re-invigoration of interventions that specifically target the socio-cognitive determinants of tobacco use, including perceptions about self-efficacy, perceived barriers, and perceived benefits of abstinence. These may have longer lasting impacts than legal restrictions and may withstand industry interference and new products. Given the unique socio-cultural dynamics of the Caribbean, these findings suggest that culturally sensitive messaging aligned with local values, language, and lived experiences could be important for increasing relevance and impact. Within Caribbean settings, recent regional sources recommend culturally relevant, locally tailored youth tobacco-prevention education aligned with community norms and language, and outline school- and community-based implementation priorities^35,36^. Culturally relevant educational interventions that highlight both personal and societal reasons for avoiding tobacco use may be worth considering for Caribbean adolescents, especially if confirmed with longitudinal studies. By aligning messages with local values and norms, such campaigns can potentially enhance engagement and effectiveness in promoting tobacco-free lifestyles. This study contributes uniquely by examining multiple HBM constructs in relation to tobacco-related beliefs among Caribbean adolescents, addressing a significant gap in regional research.

## CONCLUSIONS

The study highlights that health beliefs, including self-efficacy and perceived benefits, have declined over time and that they strongly influence Caribbean adolescents’ intentions to abstain from tobacco use. While recognizing that causal inferences cannot be drawn from cross-sectional data, our study suggests prevention strategies may benefit from emphasizing refusal skills and contextually relevant messaging. Focusing on these beliefs during adolescence could be a key area for intervention, as they are associated with tobacco-free intentions and behaviors. Future studies should employ longitudinal methodologies, so that temporal order can be established, and qualitative methods (e.g. focus groups or in-depth interviews) to capture detailed narratives of how Caribbean adolescents perceive tobacco use. Future research should also evaluate the effectiveness of targeted interventions (e.g. school-based refusal skill training or culturally tailored media campaigns) that aim to strengthen refusal self-efficacy and related beliefs among adolescents.

## Supplementary Material



## Data Availability

The data supporting this research are available from the following source: https://extranet.who.int/ncdsmicrodata/index.php/catalog/gyts/?page=1&ps=15&repo=GYTS.
