# Supplementary material for: Tobacco use perceptions and intentions to abstain among adolescents in the English-speaking Caribbean: A cross-sectional secondary analysis
Source: Tob Prev Cessat. 2026 May 30;12:10.18332/tpc/217323. doi: 10.18332/tpc/217323 (PMC13221875; doi:10.18332/tpc/217323)
Supplement: Supplementary file 1 [file TPC-12-27-s1.pdf]

Supplementary file Table 1. **Country-level differences in tobacco-related perceptions and abstinence outcomes among adolescents aged 13–15 years in three English-speaking Caribbean countries: a cross-sectional analysis of Global Youth Tobacco Survey (GYTS) data from 2000 (N = 3,431) and 2017–2018 (N = 3,767)**

**Tobacco abstinence perceptions and outcomes**	**Antigua and Barbuda**					**St Lucia**					**St. Vincent**				
		**Mean**		**95% CI**			**Mean**		**95%CI**			**Mean**		**95%CI**	
	**2000**	**2017-18**	**Diff**	**Lower**	**Upper**	**2000**	**2017-18**	**Diff**	**Lower**	**Upper**	**2000**	**2017-18**	**Diff**	**Lower**	**Upper**
Perceived Severity	0.89	0.87	-0.02	-0.06	0.02	0.89	0.83	-0.07	-0.12	-0.02	0.87	0.84	-0.03	-0.08	0.01
Perceived Benefit (friends)	0.51	0.27	-0.24	-0.29	-0.19	0.40	0.31	-0.09	-0.15	-0.03					
Perceived Benefit (attractiveness)	0.84	0.63	-0.21	-0.26	-0.17	0.81	0.66	-0.16	-0.22	-0.10	0.86	0.59	-0.27	-0.31	-0.23
Perceived Benefit (Public Bans)	0.73	0.74	0.01	-0.05	0.07	0.79	0.75	-0.04	-0.10	0.02	0.71	0.72	0.01	-0.04	0.07
Self-Efficacy	0.94	0.91	-0.03	-0.05	-0.01	0.89	0.89	0.00	-0.03	0.03	0.92	0.87	-0.05	-0.08	-0.02
Cues (Events)	0.70	0.32	-0.37	-0.42	-0.33	0.72	0.33	-0.39	-0.43	-0.34	0.69	0.27	-0.41	-0.47	-0.35
Cues (Media)	0.78	0.45	-0.33	-0.38	-0.28	0.82	0.40	-0.42	-0.46	-0.37	0.78	0.36	-0.42	-0.46	-0.38
Abstinence Intentions	0.96	0.93	-0.04	-0.05	-0.02	0.92	0.90	-0.02	-0.05	0.00	0.94	0.90	-0.04	-0.07	-0.01
Abstinence (cigarettes)	0.95	0.99	0.04	0.02	0.05	0.90	0.94	0.03	0.00	0.06	0.86	0.96	0.10	0.07	0.13
Abstinence (other)	0.90	0.95	0.05	0.02	0.07	0.93	0.96	0.03	0.01	0.06	0.87	0.94	0.07	0.04	0.10

Note: Diff = Difference. CI = confidence interval

Perceived Benefit (friends) not collected in St. Vincent

Perceived Benefit (friends) = belief that young people who smoke have fewer friends; Perceived Benefit (attractiveness) = belief that young people who smoke are less attractive; Perceived Benefit (Public Bans) = in favor of banning smoking in enclosed public spaces; Cues (Events) = See or hear ant-tobacco messages at gatherings; Cues (Media) = See or hear any anti-tobacco media messages.

© **2026 Chukwu R.O. et al.**
